# Effect of Sequential vs. Simultaneous Dual Growth Factor Release from Structured Heparin-Poly-Electrolyte Multilayer Coatings on Peri-Implant Bone Formation and Angiogenesis in Pig Mandibles

**DOI:** 10.3390/jfb16020067

**Published:** 2025-02-17

**Authors:** Philipp Kauffmann, Susanne Wolfer, Christina Behrens, Pauline Schlosser, Christian Dullin, Uwe Schirmer, Klaus Liefeith, Henning Schliephake

**Affiliations:** 1 Department of Oral and Maxillofacial Surgery, George-Augusta-University, 37075 Gottingen, Germany; philipp.kauffmann@med.uni-goettingen.de (P.K.); susanne.wolfer@med.uni-goettingen.de (S.W.); cbehren@gwdg.de (C.B.); pauline.schlosser@stud.uni-goettingen.de (P.S.); 2 Department of Diagnostic and Interventional Radiology, George-Augusta-University, 37075 Gottingen, Germany; christian.dullin@protonmail.com; 3 Department of Diagnostic and Interventional Radiology, University Hospital Heidelberg, 69120 Heidelberg, Germany; 4 Max Plank Institute for Multidisciplinary Sciences, 37077 Gottingen, Germany; 5 Institute for Bioprocessing and Analytical Measurement Techniques, 37308 Heiligenstadt, Germany; uwe.schirmer@iba-heiligenstadt.de (U.S.); klaus.liefeith@iba-heiligenstadt.de (K.L.)

**Keywords:** heparin poly-electrolyte multilayer, bone morphogenic proteins, vascular endothelial growth factor, controlled release, biofunctionalization

## Abstract

The aim of the present study was to test the sequential and simultaneous release of rhBMP2 and rhVEGF165 from poly-l-lysine-heparin (PLL-Hep) poly-electrolyte multilayer (PEM) coating on titanium surfaces for their ability to enhance peri-implant bone formation and CD31 expression around disc-shaped titanium implants (5 × 7 mm) in mini-pig mandibles. Bare titanium surfaces loaded with respective growth factor combinations served as controls. Ten different surface conditions were tested exhibiting early VEGF release, early BMP release, simultaneous VEGF and BMP release, and sole VEGF/BMP release, respectively. The implants were inserted press-fit into 5 mm trephine cavities at the lower border of the mandibles of mini-pigs and left to heal for 4 and 13 weeks. After 4 weeks, there was no significant difference in peri-implant bone formation, bone–implant contact nor CD31 expression between the different surface conditions. After 13 weeks, bone formation was significantly higher in the zone of 100 μm next to implant surfaces releasing either BMP alone or with an early release of BMP2. Expression of CD31 has significantly decreased from 4 to 13 weeks with significantly higher values in the group of implants with early release of BMP2. The results indicate that the range of released growth factors is limited to a distance of approximately 100 μm and that the sequence of early release of BMP2 followed by VEGF165 promotes peri-implant bone formation and peri-implant angiogenesis, which is in contrast to the current understanding of the temporal patterns of growth factor release for enhancement of bone formation.

## 1. Introduction

Titanium implants are widely used for the fixation of various devices to the surrounding or underlying bone. Formation of new bone tissue around the endosseous implant parts is a biological key process for the anchorage of the implant surface in viable bone. However, systemic or local conditions may deteriorate the ability of the peri-implant environment to form bone of sufficient quality or quantity to accomplish this process of osseointegration [1,2,3]. Various approaches have been used to enhance the biological quality of titanium implant surface [4,5,6,7]. One of the frequently explored avenues improving the biological interaction between the implant surface and the peri-implant bone has been the binding and release of biologically active substances such as growth factors [8,9,10,11]. Unfortunately, adsorptive coating of the bare metal surface with growth factors has been associated with a very rapid release of high dosages, leading to untoward effects with respect to osseointegration [12,13,14,15]. Strategies for more sophisticated binding and release of biologically active signals encompasses surface engineering using single layers of anchoring molecules as well as multilayer coatings of various compositions [16,17,18,19,20]. In particular, the use of poly-electrolyte multilayers employing glucosaminoglycans (GAGs) has been shown to be able to accommodate growth factors in the μg range with a release kinetic of several weeks [21,22,23]. The advantage of using GAGs as polyanions is the naturally occurring interaction with proteins, which is particularly true for heparin that additionally provides binding sites for many polypeptide growth factors involved in osteogenesis such as bone morphogenic proteins (BMPs) and vascular endothelial growth factor (VEGF) [24].

The layer-by-layer approach of multilayer film constructions on the one hand allows for the modulation of release kinetics of growth factors by additional cross-linking of the poly-electrolyte chains [21,23,25,26]. On the other hand, it can be used for a targeted incorporation of individual growth factors in different layers, thereby giving rise to a differential pattern of release in terms of sequence and dosage. Recent work has shown that variations in the architecture of poly-electrolyte multilayer coatings of poly-l-lysine (PLL) and heparin (Hep) as well as the location of growth factors within the films is important for the amount of the individual growth factor released. Thereby, the osteogenic and angiogenic properties of these modified surface variations could be modulated in a targeted manner [27,28].

It was thus the aim of the present study to assess the biological effect of an array of modifications of PLL-Hep multilayer coatings on microrough titanium surfaces loaded with osteogenic and angiogenic growth factors. Titanium discs were coated with PLL-Hep multilayer films and loaded with rhVEGF165 and rhBMP2 in different layers within the multilayer film. The square discs were inserted in a large animal model in mini-pig mandibles and evaluated for their angiogenic activity as well as for peri-implant bone formation and the degree of osseointegration.

## 2. Materials and Methods

### 2.1. In Vitro Study

#### 2.1.1. Titanium Specimen Fabrication

Square titanium discs of 7 × 5 mm (cpTi) with a thickness of 1 mm were sandblasted (MS/ERK320A, grain size 29.2 μm, CH-8181, KLS Martin, Höri, Switzerland) and acid-etched (KLS Martin, Tuttlingen, Germany) as previously described [18] in 5.1 M hydrochloric acid and 4.6 M sulphuric acid solution for 300 s at 108 °C. The discs had a trapezoid profile to provide a sharp cutting edge on both sides for press-fit insertion into trephine holes (see below).

#### 2.1.2. Multilayer Coating of Ti Discs

The Ti-specimens were coated with a heparin-based poly-electrolyte multilayer film (PLL-Hep-PEM) for growth factor loading later on. Poly-L-lysine (PLL, 30–70 kDa, Sigma Aldrich, Taufkirchen, Germany) and heparin (Hep, 50 mg/mL, from porcine intestinal mucosa) were assembled as the initial double layer on the metal surface. Subsequently, nine double layers of collagen I (rat tail collagen type I, ibidi, Gräfeling, Germany, 5 mg/mL) and heparin were added to the surface resulting in a (PLL-Hep1) (Col-Hep9) poly-electrolyte multilayer film, referred to as Col-Hep PEM.

For fabrication of PEM films, poly-l-lysine (30–70 kDa) and heparin (50 mg/mL), from porcine intestinal mucosa, (Sigma-Aldrich (Taufkirchen, Germany)) were used without further purification. The poly-electrolytes were dissolved in 5 mM acetate at a concentration of 1 mg/mL. Film construction was performed semi-automatically employing a dipping robot (DR3, Riegler&Kirstein, Potsdam, Germany). The cleaned Ti specimens were first soaked into the PLL solution for 5 min followed by three washing steps in deionized water to remove unbound PLL. Subsequently, heparin adsorption was performed in an identical fashion. Dipping cycles were repeated until the desired numbers of double layers and film architecture, respectively, were achieved. All samples were rinsed in deionized water and air-dried in a gentle stream of pressurized air.

The resulting films had been characterized in previous reports [28,29] using Quartz crystal microbalance, atomic force microscopy, SEM, and profilometry to assess surface structure, film growth, and topography of the film systems. The (PLL-Hep)10 multilayers on the titanium surfaces had a total mass of 6.33 ± 0.32 μg/cm^2^ with a linear growth to a thickness of 63.4 ± 3.21 nm indicating a dense and homogenous structure. Surface roughness (Ra, Sa) of the different specimens varied between Ra: 3.41 μm, Sa: 3.62 μm for the uncoated Ti surfaces, Ra: 3.08 μm, Sa: 3.04 μm for the Ti surfaces coated with a (PLL-Hep)10 multilayer film, and Ra: 2.81 μm, Sa: 3.38 μm for the (PLL-Hep)10 PEM loaded with rhBMP2. Differences in surface roughness between the three different surfaces were not significant. For more details, the reader is referred to the above-mentioned reports [28,29].

#### 2.1.3. Variation in Film Architecture and Growth Factor Loading

Five combinations of film architecture and loading patterns with rhBMP2/rhVEGF165 were produced on the Ti discs:

(a) One (PLL-Hep)_20_ multilayer system that was loaded with rhBMP2 (20-rhBMP2).
(b) One (PLL-Hep)_20_ multilayer system that was loaded with rhVEGF165 (20-rhVEGF165).
(c) Two subsequent (PLL-Hep)_10_ multilayer systems for dual growth factor loading using a two-step procedure: a (PLL-Hep)_10_ multilayer system was loaded with one growth factor, after which a second (PLL-Hep)_10_ multilayer system was added with subsequent loading of the second growth factor on top (10-rhBMP2-10-rhVEGF165).
(d) This procedure was modified by changing the sequence of growth factor loading (10-rhVEGF165-10-rhBMP2).
(e) One (PLL-Hep)_20_ multilayer system that was loaded with both growth factors together (20-rhBMP2 + rhVEGF165).

Titanium discs with unloaded (PLL-Hep)_20_ multilayer coatings as well as uncoated bare Ti discs with adsorptive loading with a single growth factor (rhBMP2, rhVEGF165) and two growth factors (rhBMP2 and rhVEGF165) served as controls. This resulted in 10 different surface conditions to be tested (Table 1). Discs were evaluated in vitro for the amount of growth factor incorporated and released and in vivo for the amount of peri-implant bone formation and the degree of osseointegration. Moreover, expression of CD31 was evaluated as angiogenic marker using immunofluorescence microscopy.

#### 2.1.4. Growth Factor Loading/Samples for In Vitro Release

Three-dimensional-printed silicone mounts were used as containers for the titanium discs in groups of three discs. The containers exposed only one side of the disc and were dipped overnight in 160 μL of a loading solution with either rhBMP2 (75 μg/mL; Chinese Hamster Ovary cell-derived, PeproTech, Hamburg, Germany) or rhVEGF165 (75 μg/mL; Human Embryonic Kidney 293 cell-derived, ThermoFischer, GIBCO, Darmstadt, Germany), corresponding to 3 μg per specimen. The discs with dual growth factor loading were first incubated with rhVEGF165 followed by rhBMP2. The concentrations of growth factors in the loading solutions for rhBMP2 and rhVEGF165 had been defined during previous experiments [28].

For the in vivo experiments, the PEM-coated and uncoated titanium discs were individually loaded with growth factors on both sides. The samples were deposited into a single well each with 3D-printed silicone containers and dipped overnight in 80 μL of loading solutions with rhBMP2 (75 μg/mL; Chinese Hamster Ovary cell-derived, PeproTech, Hamburg, Germany) or rhVEGF165 (75 μg/mL; Human Embryonic Kidney 293 cell-derived, ThermoFischer, GIBCO, Darmstadt, Germany), corresponding to 6 μg growth factor per specimen reservoir. The discs with dual growth factor loading were first incubated with rhVEGF165 followed by rhBMP2. After loading has been completed, the discs were washed twice with deionized water and shortly air-dried at RT. The fully prepared discs were stored at 4 °C.

Loading efficacy for rhBMP-2 and rhVEGF165 was assessed indirectly by measuring the remaining amount of both growth factors in the stored supernatant of the coating procedure. A Bicinchoninic Acid (BCA) Protein Assay Kit (ThermoScientific, Darmstadt, Germany) with bovine serum albumin (BSA) as standard was applied using 25 μL of each standard (working range 25–2000 μg/mL or 5–250 μg/mL). The samples were replicated into a microplate well (96-well plates) and after addition of 200 μL of working solution to each well, the plates were placed on a plate shaker for 30 s (37° C, 400 rpm, THERMOstar, BMG LABTECH, Ortenberg, Germany) and incubated at 37 °C for 30 min. Absorbance at 562 nm was measured with an ELISA plate reader (SpectraMax M2, Molecular Devices, San Jose, CA, USA) at room temperature (RT).

When subsequent loading with two growth factors was performed, possible eluation of the first loaded growth factor during the second loading procedure was tested using the BMP2 or VEGF using enzyme-linked immunosorbent assay (ELISA), respectively, as described below. Only 0.06–0.89% of the initially loaded amount of the first growth factor could be detected. Thus it can be assumed that the coating and loading procedure of the second zone and growth factor was associated with only minimal leaching of the first loaded factor.

#### 2.1.5. Release Experiments

Experimental procedures have been described before in detail [28]. In brief, growth factor-loaded specimens were incubated in 250 μL DMEM supplemented with 2% FCS and 1% penicillin/streptomycin at 37 °C in 24-well plates with medium being collected and replaced after 24, 48, 72 h and every 3 days thereafter until day 21. Release profiles of rhBMP2 and rhVEGF165 were assessed using Human/Murine/Rat BMP2 and Human VEGF Standard TMB ELISA Development Kit (PeproTech, Hamburg, Germany), respectively, according to the instructions of the supplier. RhBMP2 (PeproTech, Hamburg, Germany) or rhVEGF-165 (ThermoFisher, Gibco, Waltham, MA, USA) were used as standard. An ELISA plate reader (SpectraMax M2, DE 8377 Munich, Germany) was used at 450 nm with wavelength correction set at 620 nm. All measurements were performed twice on three titanium discs each.

### 2.2. In Vivo Study

#### 2.2.1. Sample Size Calculation

The ten different surface conditions were planned to be evaluated after 4 and 13 weeks each. In order to maintain a Family-Wise-Error-Rate of 5% across the comparisons, each comparison was performed at a level of significance of α = 5%/14 = 0.36%. A detectable difference in bone formation/degree of osseointegration was assumed to be relevant at an increase by 10% starting from 10% with a standard deviation of 10% per interval/surface condition. In order to detect this increase as significant at an assumed standard deviation of σ = 10% within the groups at a significance level of α = 0.36% and a power of 1-β = 80%, a group size of 6 animals was calculated.

#### 2.2.2. Surgical Procedures and Animal Care

A mini pig model was chosen as the bone biology in these animals compares well with human biology [30]. According to the sample size calculation, 12 animals were used (gender: female; age: 2–3 y, weight 44.6 ± 7.6 kg). The animals were randomly allocated to two groups of six animals each for evaluation after 4 weeks and 13 weeks, respectively. All surgical procedures, housing, and animal care were carried out in accordance with the German legislation for animal protection and the regulations for animal experiments of the state of Lower Saxony. Ethical clearing has been obtained under the license number 20/3554. Animals were held in groups of 2–3 animals in cages with concrete floor with sawdust bedding and wooden walls. Prior to the start of the surgical procedures, the animals were allowed to accommodate for four weeks. All animals presented in good health. All procedures were performed in the animal facilities of the University Medicine Goettingen following the ARRIVE guidelines [31]. The experiments were conducted between 02/2022 and 06/2022. Sedation was initiated by an orally administered dose of 0.5 mg/kg body weight of Diazepam followed by intramuscular injection of 10 mg/kg body weight Ketamine and 2 mg/kg body weight Azaperone after approximately 20 min. General anesthesia was induced with titrated i.v. administration of Thiopental followed by endotracheal intubation. Anesthesia was maintained using 2–4% of Isoflurane, supported by Piritramid and Ketamine to add analgesic capacity. A Dexpanthenole lotion (Bepanthen©, Bayer AG, 51368 Leverkusen, Germany) was used to cover the eyes.

**Figure 1:**
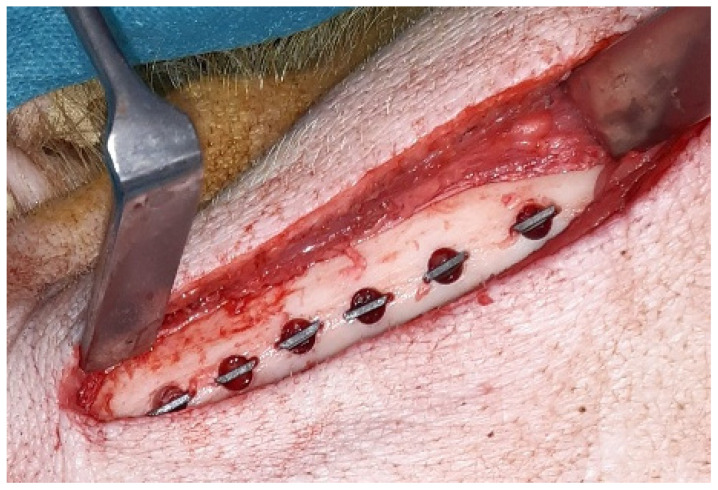
Clinical picture of implant insertion into the lower border of a mini pig mandible:.

A bilateral submandibular approach was chosen for surgical access to the mandible. Trephine holes of 5 mm diameter and 5 mm depth were created at the lower border of the mandible with vertical bone cuts of 0.5 mm depth at the mesial and distal side created with a fissure burr (Figure 1). The setting included four more groups with different experimental surface conditions that will be reported elsewhere [32]. The discs were placed press-fit into the trephine cavities. The allocation of the individual position of the different surface modifications to the trephine drill holes along the mandibular border was defined by drawing lots. Subsequently, the periosteum overlying the opening of the cavity was removed to exclude bias in bone formation due to excessive periosteal bone regeneration and wounds were closed in layers using resorbable sutures (Vicryl 3.0, Ethicon, Norderstedt, Germany).

During the first postoperative week, animals were visited twice per day. Analgesic medication (0.6 mg Buprenorphin with 5 mg/kg body weight Carprofen) was administered intravenously in the first three days. At any signs of discomfort, 5–7.5 mg/kg body weight Carprofen were administered additionally per os.

#### 2.2.3. Histologic Preparation and Morphometry

After 4 weeks and 13 weeks, the mandibles of 6 animals each were retrieved and the implanted discs with surrounding bone were removed using a diamond saw (EXAKT ©, Robert-Koch-Str. 5, DE-22851 Norderstedt, Germany) followed by dehydration and embedding into Technovit 9100 © (Heraeus Kulzer GmbH, Philipp-Reis-Str. 8/13, 61,273 Wehrheim, Germany). Thick-section specimens [33] were produced from each disc and its surrounding bone in a plane parallel to the edge of the longer side of the discs starting from the lower border of the mandible in upward direction. The resulting specimens were surface stained using both Toluidin Blue and Alzarine-Methylene Blue. For histomorphometry, the trephine defect area as a whole was evaluated separately from the area adjacent to the implant surface. The peri-implant zone was defined as a 300 μm thick layer and was divided into 3 layers of 100 μm thickness each on both sides of the implant cross-section, creating an (i) immediate, (ii) intermediate, and (iii) remote tissue layer in relation to the implant surface. Each layer was divided into three sections: (i) one central third and (ii) two peripheral thirds next to the wall of the trephine cavity on both sides. This resulted in 18 distinct areas of peri-implant tissue allowing to assess the effect of distance from the implant surface and distance from the cavity walls on the efficacy of released growth factors on peri-implant bone formation and bone anchorage.

For morphometric evaluation, specimens were scanned using a digital scanning device (Dotslide-System2.0 **©**, Olympus Deutschland GmbH, Wendenstraße 14–18, 20097 Hamburg, Germany). The resulting digital image data were analyzed using a custom-made Python3-based image analysis pipeline utilizing the common Python modules scikit-image, matplotlib, opencv, and pandas.

**Figure 2:**
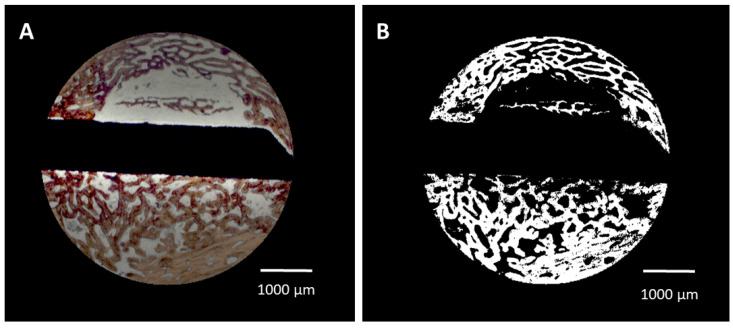
(**A**) Selection of the trephine defect for morphometric evaluation and (**B**) digitization of the bone area.

Primary outcome parameters were:

(i). Bone area/bone density. The algorithm automatically identified the color of the Alizarine Red-stained areas in the cross-section specimens and assessed the area occupied by bone both in absolute values (bone formation (BF)) and in relation to each section area (bone density (BD)) by pixel counting. Pixels were converted in mm^2^ using the calculated pixel size of 17.43 μm^2^/pixel (Figure 2A,B). Bone density was only evaluated for the trephine defects as a whole. To account for variations in the appearance of the color of Alizarine Red in the difference and in cases in which the newly formed bone covered the entire trephine defect, parameters were manually adjusted.
(ii). Bone–implant contact (BIC). The algorithm identified the surface area occupied by bone by image analysis routines and calculated the bone–implant contact (BIC) as percentage of occupied surface area. In brief, the surface of the identified cross-section of the implant was enlarged by 1 pixel (approx. 4.18 μm) and limited to the trephine defect size. The resulting mask was multiplied with the bone mask and the ratio of those pixels to the entire surface of the implant was calculated.

Measurements were performed by one blinded examiner, who was calibrated during introduction to the image analysis system. Outcome parameters were assessed individually for each cross-section; mean values were calculated for each disc from 3–4 cross-sections.

#### 2.2.4. Immunohistochemical Preparation and Evaluation of Immunofluorescence

Technovit embedded thick-section specimens (see Histologic Preparation and Morphometry) were mounted on glass slides (Paul Marienfeld GmbH, Lauda-Koenigshofen, Germany). After preparation of tissue sections with a resulting thickness of 70–100 μm, the sections were incubated three times with xylene, 20 min each, and placed three times (twice for 20 min and once overnight) in MEA (2-methoxyethylacetate, Merck, Darmstadt, Germany). The specimens were then rehydrated in descending concentrations of ethanol (100%, 96%, 70%, twice for 2.5 min each) and washed twice in deionized water for 2.5 min each.

For the immunofluorescence staining, the deplasticized and rehydrated bone tissue sections were incubated in 1× citrate-based TR buffer, pH 6.0 (Target Retrieval Solution, Agilent Dako, Waldbronn, Germany) for 30 s at 121 °C followed by incubation for 10 s at 90 °C using a standard pressure cooker (PASCAL S2800, Dako, Hamburg, Germany). Subsequently, the sections were incubated for 5 min at room temperature (RT), washed for 10 min in deionized water, and lastly, washed three times in PBS for 5 min each. Next, the samples were incubated for 1 h at RT in blocking buffer (10% goat Serum Block in PBS, Histoprime Biozol, Eching, Germany). For the detection of CD31, the specimens were incubated with an anti-CD31 antibody (CD31/PECAM1, Platelet/endothelial cell adhesion molecule-1, ABIN 726140, 1:100; antikoerper-online.de, Aachen, Germany) at 4 °C overnight. After washing three times in PBS, 5 min each, the specimens were labeled using the secondary antibody Alexa Fluor 647 (ab 150079, 1:500; Abcam, Cambridge, UK) by incubation for 1 h at RT followed by washing three times in PBS for 5 min each. Subsequently, nuclei were counterstained with DAPI (1:1000; Sigma-Aldrich Merck, Darmstadt, Germany) for 10 min at RT. Finally, the sections were washed in PBS (three times, 5 min each) and mounted with Fluor Save Reagent (30 min at RT and overnight at 4 °C; Merck Millipore, Darmstadt, Germany).

The antibodies and DAPI were diluted using Antibody Diluent (Agilent Dako, Waldbrunn, Germany). All incubations, including blocking, were performed in a humidity chamber.

**Figure 3:**
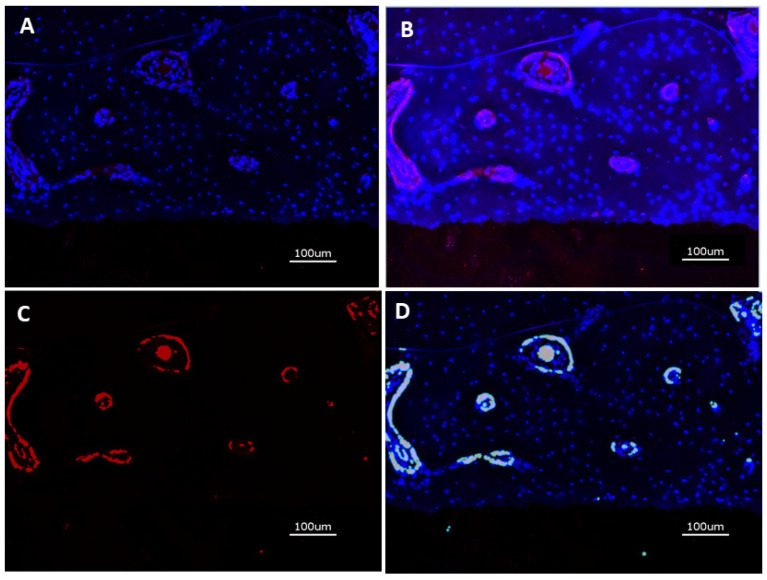
(**A**): DAPI stain of nuclei in peri-implant tissue (blue); (**B**) Overlay of DAPI stain and CD31 expression (red); (**C**) Isolation of CD31-positive area; (**D**) Digitization of CD31-positive area.

After immunostaining, the specimens were analyzed with the KEYENCE BZ-X710 microscope (Keyence, Neu Isenburg, Germany) using a Cy5 filter (OP-87766) Keyence, Neu Isenburg, Germany) for detection of the target protein CD31 and the DAPI filter (OP-87762) (Keyence, Neu Isenburg, Germany). Sections stained without the primary antibody served as controls. One cross-sectional specimen of each surface condition per animal was evaluated. Quantitative analysis was performed on digital images of the specimens at 20-fold magnification corresponding to an image size of 725 × 543 μm displaying the implant surface at the lower border of the field of view and peri-implant tissue up to a distance of approximately 400 μm thickness. Thirteen images, placed on the upper (7) and lower (6) edge of the cross-sections were analyzed. Quantification of CD31 expression was measured in μm^2^ using the BZ-X-Analyzer tool filter for red light; overlay images with DAPI-stained nuclei were used for analysis (Figure 3A–D).

#### 2.2.5. Statistics

Data are presented as means ± standard deviation (SD). Shapiro–Wilk tests had shown that the results of all surface conditions had a non-normal distribution (*p* ≤ 0.001). Therefore, Friedman tests (SPSS Statistics 26.0, https://www.ibm.com/spss) with Bonferroni correction were used to compare bone formation (BF), bone density (BD), and bone–implant contact (BIC) as well as expression of CD31 between the experimental surfaces. Bone density was only assessed in the evaluation of the whole trephine defect as this parameter is of little significance in a the 100 μm thick layers considered separately. Moreover, the effect of healing time was assessed by comparing the two intervals using Mann–Whitney tests and a possible correlation between the amount of bone formation and the bone contact rate of the implant surface as well as the expression of CD31 which was evaluated using Spearman correlation tests. All tests were performed at a significance level of 5%.

## 3. Results

### 3.1. In Vitro Experiments

#### 3.1.1. Growth Factor Loading

Loading of the (PLL-Hep)_20_ films with rhVEGF165 and rhBMP2 alone lead to the incorporation of comparable of 4.4 μg (SD 2.0) VEGF and 5.7 μg (SD 0.4) BMP per cm^2^ surface, respectively (Table 1). Loading of the (PLL-Hep)10 films with rhBMP2 first and subsequent build-up of an additional (PLL-Hep)10 films followed by loading with rhVEGF165 resulted in the incorporation of less rhBMP2 (3.8 μg (SD 1.3)) and more rhVEGF165 (5.5 μg (SD 0.4)). When the sequence was swapped with rhVEGF165 in the lower film system and rhBMP2 in the upper, comparable amounts of growth factors were loaded into both film systems (rhBMP2: 5.9 (SD 0.6); rhVEGF165: 5.0 (SD 0.7). Loading of rhBMP2 and rhVEGF165 in the same (PLL-Hep)_20_ film resulted in lower loading of rhBMP2 (3.5 μg (SD 1.5) than rhVEGF165 (4.5 μg (SD 2.1). Loading of bare Ti surfaces with rhBMP2 alone resulted in lower amounts of growth factor bound to the surface when compared to the PLL-Hep film-coated discs whereas loading with rhVEGF resulted in adsorption of a comparable amount of growth factor as loading of (PLL-Hep)_20_ films.

#### 3.1.2. Growth Factor Release

During the 21-day interval of in vitro release, the films with a 2-zone architecture with sequential loading of rhVEGF165 and rhBMP2 into (PLL-Hep)_10_ films each released minimal amounts of rhBMP2 when it was incorporated into the lower zone (0.06 μg/cm^2^ surface, SD 0.01), compared to the release from the same films loaded with rhBMP2 alone (2.59 μg, SD 0.43) (Table 2). In contrast, the release of rhVEGF165 from the upper layer of the 2-zone architecture was higher than from of the (PLL-Hep)_20_ films loaded with VEGF alone (2.63 μg, SD 0.27 vs. 1.70 μg, SD 0.28). This was reversed when the location of the growth factors was swapped with a slightly increased release of rhBMP2 from the upper zone of 3.03 μg (SD 0.47) compared to loading with rhBMP2 alone (2.59 μg, SD 0.43), whereas the delivery of rhVEGF165 was almost completely suppressed (0.01 μg, SD 0.001) during the observation period. When both growth factors were loaded together into one (PLL-HEP)_20_ film, the release of rhBMP2 and rhVEGF165 was at an equal level of 1.96 μg (SD 0.28) and 1.49 μg (SD 0.29), respectively. Release from the bare metal surfaces rhBMP2 and rhVEGF165 loading alone produced 0.27 μg VEGF (SD 0.02) and 0.36 μg BMP (SD 0.03). Discs with combined loading with both growth factors released 0.89 μg rhBMP2 (SD 0.66) and 0.16 μg of rhVEGF165 (SD 0.05) on average during the observation period.

### 3.2. In Vivo Experiments

Postoperative healing was uneventful. No animal was removed from the evaluation. Analgesic therapy could be stopped after postoperative 5 days. All 12 animals were included in the evaluation. During the preparation of the specimens, it became obvious that one disc with a (PLL-Hep)_20_ multilayer system that was loaded with rhVEGF165 only had been lost. All other 167 discs could be submitted to the scheduled morphologic and quantitative evaluation.

#### 3.2.1. Histology

**Figure 4:**
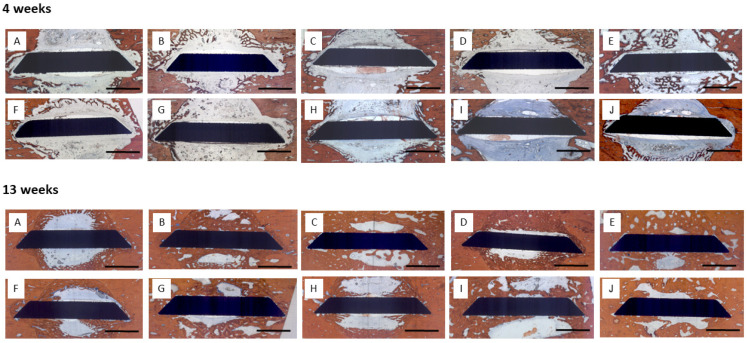
Micrographs of peri-implant bone formation: (**Top Row**): 4 weeks, (**Bottom Row**): 13 weeks, Bar: 2000 μm; (**A**) (PLL-Hep)**_10_** multilayer system loaded with rhBMP2 with a second (PLL-Hep)_10_ multilayer system loaded with rhVGEF_165_ on top; (**B**) (PLL-Hep)_10_ multilayer system loaded with rhVGEF_165_ with a second (PLL-Hep)_10_ multilayer system loaded with rhBMP2 on top; (**C**) (PLL-Hep)_20_ multilayer system simultaneously loaded with rhVGEF_165_ and rhBMP2; (**D**) (PLL-Hep)_20_ multilayer system loaded with rhVGEF_165_ only; (**E**) (PLL-Hep)_20_ multilayer system loaded with rhBMP2 only; (**F**) (PLL-Hep)_20_ multilayer system without growth factor loading (control); (**G**) Ti surface loaded simultaneously loaded with rhVGEF_165_ and rhBMP2; (**H**) Ti surface loaded with rhVGEF_165_ only; (**I**) Ti surface loaded with rhBMP2 only; (**J**): Ti surface unloaded (control).

Four weeks: Little bone formation was observed after 4 weeks originating from the trephine defect walls (Figure 4). Sparse bone trabeculae were seen in contact with the surface of the disc implants. In some defects, trabecular bone formation had occupied more volume; however, this was not associated with a specific coating or growth factor loading.

Thirteen weeks: Substantially increased bone formation was seen after 13 weeks that almost completely filled the defect volume in some specimens, again without being associated with a certain surface condition or growth factor loading. The predominant pattern of immediate peri-implant bone regeneration was characterized by a propagation along the implant surfaces with bone formation originating from the defect walls (Figure 4). Surfaces with early release of BMP-2 exhibited a higher degree of osseointegration with more complete coverage of the implant surface by newly formed bone.

#### 3.2.2. Histomorphometry

##### 4 Weeks

**Figure 5:**
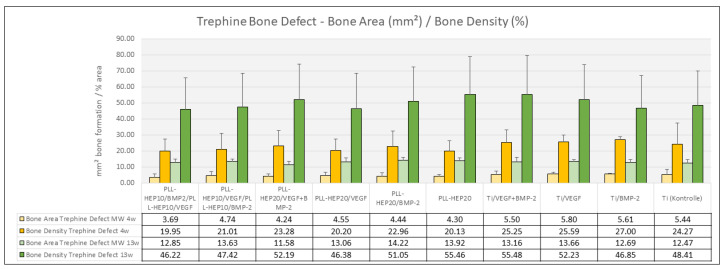
Bone area (mm^2^) and bone density (%) of newly formed bone within the trephine defects. Data are presented as means ± standard deviation (SD) with *n* = 6.

**Figure 6:**
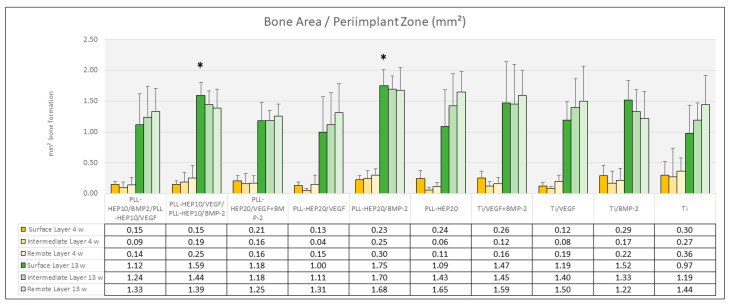
Bone area (mm^2^) of newly formed bone within the peri-implant zone (300 μm). Data are presented as means ± standard deviation (SD) with *n* = 6; (*): significantly different from Ti control surface (*p* ≤ 0.05).

**Figure 7:**
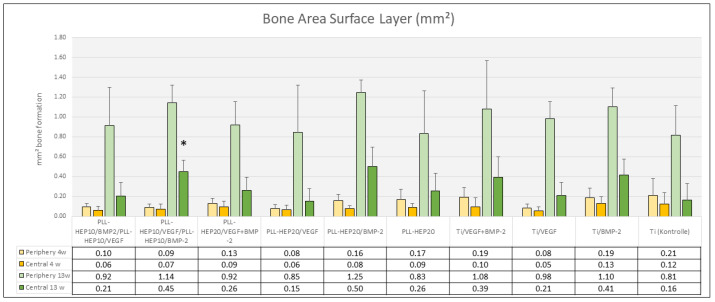
Bone area (mm^2^) of newly formed bone within the immediate surface layer (100 μm). Data are presented as means ± standard deviation (SD) with *n* = 6. (*): significantly different from Ti control surface (*p* ≤ 0.05).

***Bone formation:*** After 4 weeks, bone formation in the trephine defects ranged between 4.23 mm^2^ ((PLL-Hep)_20_/VEGF + BMP2) and 5.79 mm^2^ (uncoated Ti discs loaded with VEGF) with no significant differences between the experimental surface conditions and the controls (*p* = 0.733) (Figure 5). Bone density varied between 18.3% (2-zone architecture with VEGF on top and BMP below) and 26.8% (uncoated Ti discs, loaded with BMP) without significant differences (*p* = 0.676). Bone regeneration in the three peri-implant zones (immediate, intermediate, and remote layer) was considerably lower compared to the whole defect volume with amounts of bone formation varying between 0.04 mm^2^ and 0.36 mm^2^ and bone density ranging between 2.5 and 16.5%. As with the trephine defects as a whole, no significant differences were found in the individual zones between the multilayer coatings loaded with BMP with or without VEGF and the respective control surface conditions (*p* = 0.090, *p* = 0.090, *p* = 0.483, respectively) (Figure 6). The same held true when the central parts and the peripheral parts of the peri-implant zone immediately to the implant surface were considered (*p* = 0.079 and 0.285, respectively) (Figure 7).

**Figure 8:**
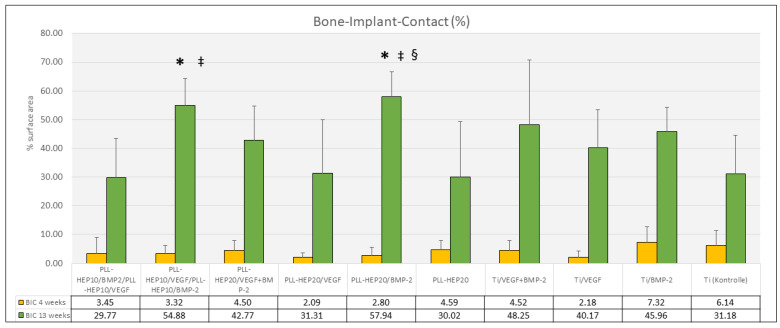
Bone–implant contact (%); data are presented as means ± standard deviation (SD) with *n* = 6; (*): significantly different from Ti control surface (*p* < 0.05); ^‡^: significantly higher than PEM coating with the 2-zone architecture with VEGF in the upper zone and BMP below as well as PEM coating with VEGF loading only (*p* << 0.05); (**^§^**): significantly different from PEM-coated unloaded surface (*p* < 0.05).

***Bone–implant contact:*** Bone–implant contact after 4 weeks showed considerable variation between the different surface groups ranging between 2.1% ((PLL-Hep)_20_ with VEGF loading) and 7.1% (uncoated Ti discs loaded with BMP) (Figure 8). No significant differences were found between the multilayer coating loaded with growth factors and the respective controls (*p* = 0.741). There was a significant correlation between bone–implant contact and newly formed bone area in the surface layer next to PEM-coated and non-coated implants loaded with VEGF and with BMP2 (*p*-values ranged between 0.005 and 0.042) (Table 3).

##### 13 Weeks

***Bone formation:*** After 13 weeks, the area of newly formed bone had significantly increased in all sections of evaluation (*p*-values varied between 0.041 and 0.002 for the different sections). Area of new bone formation in the trephine defects ranged between 11.6 mm^2^ (2-zone architecture with BMP on top and VEGF below) and 13.7 mm^2^ (uncoated Ti discs, loaded with VEGF) (Figure 5) without significant differences between the surface conditions (*p* = 0.466). The same held true for bone density of the regenerated bone within the trephine defects (*p* = 0.381) with values ranging between 56.2% ((PLL-Hep)_20_ loaded with VEGF) and 64.3% ((PLL-Hep)_20_ loaded with BMP) (Figure 5). When the three peri-implant zones were considered, the average area of newly formed bone was not significant different between the various surface conditions in the intermediate and the remote layer. When the immediate surface layer was analyzed, the largest area of bone formation in this zone was found in the group of implants coated with the (PLL-Hep)_20_ loaded with BMP (1.75 mm^2^) and implants with a 2-zone (PLL-Hep)_10_ architecture with VEGF in the lower zone and BMP in the upper zone (1.59 mm^2^). Mean values of these two groups were significantly higher than the uncoated titanium controls (*p* = 0.013 and *p* = 0.017, respectively), whereas the remaining surface modifications failed to show significant differences compared to the uncoated/unloaded Ti controls (Figure 6). When the immediate peri-implant zone next to the implant surface was considered separately for the central and the peripheral parts of the implant surface, a significant difference in bone formation between the different surface conditions was found only in the central (*p* = 0.016) but not in the peripheral parts (*p* = 0.186). In the central part, pairwise comparison with Ti controls showed a significant increase in bone formation only for implants coated with a 2-zone (PLL-Hep)_10_ architecture with VEGF in the lower zone and BMP in the upper zone (*p* = 0.022) (Figure 7).

***Bone–implant contact:*** Bone–implant contact after 13 weeks showed corresponding results to the amount of bone formation in the immediate surface layer. The highest rate of bone–implant contact was found in the groups of implants coated with (PLL-Hep)_20_ loaded with BMP (57.9%), the lowest in implants with a 2-zone (PLL-Hep)_10_ architecture with BMP in the lower zone and VEGF in the upper zone (29.8%) (*p* = 0.046) (Figure 8). Pairwise comparison showed significant differences between the uncoated/unloaded Ti controls and implants coated with (PLL-Hep)_20_ loaded with BMP (*p* = 0.013) and implants coated with a 2-zone (PLL-Hep)_10_ architecture with VEGF in the lower zone and BMP in the upper zone (*p* = 0.017). Moreover, these two surface conditions presented significantly higher mean value than the PEM-coated 2-zone architecture with VEGF in the upper layer (*p* = 0.006 and *p* = 0.004, respectively) and PEM-coated surfaces with VEGF loading alone (*p* = 0.036 and *p* = 0.028, respectively). Implants coated with (PLL-Hep)_20_ loaded with BMP only exhibited also a significantly higher BIC than implants with (PLL-Hep)_20_ loaded without growth factor loading (*p* = 0.045). All other surfaces were not significantly different from the uncoated/unloaded controls. The correlation between bone–implant contact and newly formed bone area in the surface layer after 13 weeks was significant for implants with PEM coating and with VEGF and with BMP as well as with the 2-zone architecture with BMP on the upper zone. Moreover, uncoated implants with VEGF and BMP loading and with VEGF loading alone exhibited a significant correlation (*p*-values ranged between 0.001 and 0.042) (Table 3).

#### 3.2.3. Immunofluorescence of CD31 Expression

**Figure 9:**
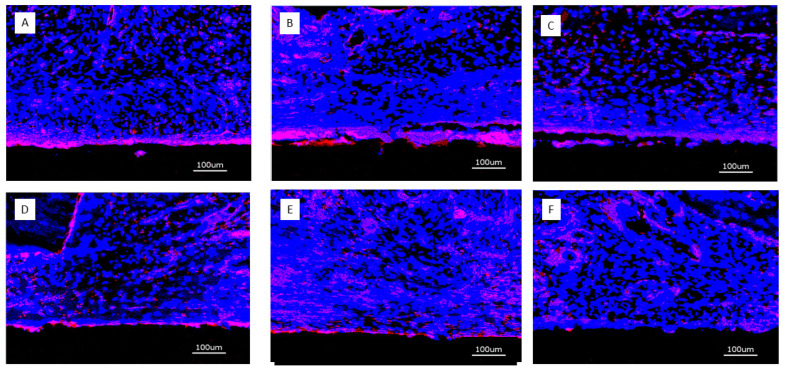
Overview of CD31-positive expression after 4 weeks (bar = 100 μm); (**A**) (PLL-Hep)**_10_** multilayer system loaded with rhBMP2 with a second (PLL-Hep)_10_ multilayer system loaded with rhVGEF_165_ on top; (**B**) (PLL-Hep)**_10_** multilayer system loaded with rhVGEF_165_ with a second (PLL-Hep)_10_ multilayer system loaded with rhBMP2 on top; (**C**) (PLL-Hep)_20_ multilayer system simultaneously loaded with rhVGEF_165_ and rhBMP2; (**D**) Ti surface loaded simultaneously loaded with rhVGEF_165_ and rhBMP2; (**E**) Ti surface loaded with rhBMP2 only; (**F**) Ti surface unloaded (control).

Four weeks: After 4 weeks, CD31 expression was strongest immediately adjacent to the implant surface of implants loaded with growth factors. There was no clear distinction between surfaces releasing BMP2, VEGF165, or both. The soft tissue overlying the implant surface exhibited a diffuse distribution of CD31 expression across the captured thickness (Figure 9). Titanium controls did not show the stronger positivity of CD31 expression immediately adjacent to the implant surface.

**Figure 10:**
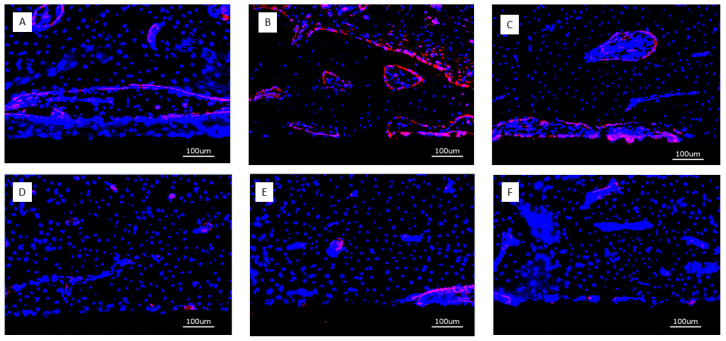
Overview of CD31-positive expression after 13 weeks (bar = 100 μm); (**A**) (PLL-Hep)**_10_** multilayer system loaded with rhBMP2 with a second (PLL-Hep)_10_ multilayer system loaded with rhVGEF_165_ on top; (**B**) (PLL-Hep)**_10_** multilayer system loaded with rhVGEF_165_ with a second (PLL-Hep)_10_ multilayer system loaded with rhBMP2 on top; (**C**) (PLL-Hep)**_20_** multilayer system simultaneously loaded with rhVGEF_165_ and rhBMP2; (**D**) Ti surface loaded simultaneously loaded with rhVGEF_165_ and rhBMP2; (**E**) Ti surface loaded with rhBMP2 only; (**F**) Ti surface unloaded (control).

Thirteen weeks: Expression of CD31 was much more confined to structural elements of the peri-implant tissues such as perivascular tissue or osteoid seams lining bone trabeculae. Implants with bare titanium surface showed only sparse positivity for CD31 (Figure 10).

#### 3.2.4. Histomorphometry of Immunofluorescence of CD31 Expression

**Figure 11:**
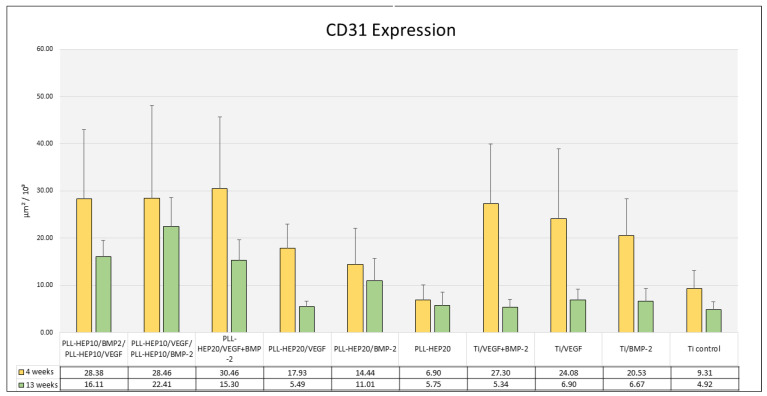
Area of CD31-positive expression (μm^2^/10^3^); data are presented as means ± standard deviation (SD) with n = 6.

Four weeks: After 4 weeks, the area of expression of CD31 varied between 6.9 and 30.5 × 10^3^ μm^2^ (Figure 11). Differences between the individual surface conditions were highly significant (*p* = 0.004). Discs with a 2-zone architecture with BMP on top and VEGF below and vice versa as well as discs with dual growth factor loading exhibited the highest mean values. Only the 2-zone PEM films with BMP loading in the upper zone and VEGF loading below exhibited mean values that were significantly different from unloaded PEM films (*p* = 0.026) (Figure 11). Differences between discs with PLL-Hep coating and growth factor loading and discs with growth factor-loaded bare Ti surfaces were not significant.

Thirteen weeks: After 13 weeks, the expression of CD31 in peri-implant tissues had decreased for all surfaces resulting in a range between 4.9 and 22.4 × 10^3^ μm^3^ (Figure 9). This decrease from 4 weeks to 13 weeks was significant for all discs with growth factor-loaded bare Ti surfaces (*p* = 0.002 for VEGF-BMP as well as BMP-only loading and *p* = 0.009 for VEGF-only loading). Moreover, discs with PLL-Hep coating with early VEGF release (2-zone architecture with VEGF on top and BMP below) or VEGF-only release exhibited a significant decrease (*p* = 0.041 and 0.004, respectively). The highest mean values have been found in the group of discs with a 2-zone architecture with BMP on top and VEGF below, which was significantly higher than the unloaded PEM films (*p* = 0.007), uncoated Ti surface with single (BMP: *p* = 0.027) and dual (VEGF and BMP: *p* = 0.001) growth factor loading as well as unloaded Ti controls (*p* = 0.007). Differences between uncoated Ti surfaces with growth factor loading exhibited no significant differences when compared to the unloaded control Ti surface.

Both after 4 and after 13 weeks, a significant correlation was found between the expression of CD31 and the area of newly formed bone in implants with a 2-zone PEM coating with BMP in the upper zone. (*p* = 0.042 and *p* = 0.019, respectively) (Table 3).

## 4. Discussion

The present study has assessed the effect of dual growth factor loading of a poly-electrolyte multilayer (PEM) coating of titanium implants on peri-implant bone formation and bone–implant contact as well as the level of angiogenic activity. Heparin had been chosen as polyanion as it is a naturally occurring component of the intercellular matrix and a large number of polypeptide growth factors provide binding sites for heparin [24]. Previous in vitro work had shown that variations in heparin-PEM film architecture with differential loading of rhVEGF165 and rhBMP2 had been able to modify the angiogenic and osteogenic properties in a targeted way [28]. In the present study, the in vitro pattern of release of BMP and VEGF had confirmed that the 2-zone architecture has produced an early angiogenic/osteogenic activity of the experimental surfaces depending on the growth factor located in the upper zone, whereas the growth factor in the lower zone did not contribute to the biological activity during this early period. A simultaneous combination of both angiogenic and osteogenic activity was only achieved when the films were loaded with BMP and VEGF together. The idea behind this approach has been to evaluate the effect of early angiogenic vs. osteogenic activity independently by delivery of the superficially located growth factor during the initial period of bone healing followed by a late release of the complementary activity during continuing eluation or degradation of the PEM films and to compare this sequence with a continuous combined angiogenic and osteogenic activity early on. The modification of the temporal pattern of release from multilayers loaded with multiple growth factors has been approached previously using barrier layers of biomimetic Ca-Phosphate [34,35] or intervening layers of poly-acrylic acid, laponit, or chitosan [35,36,37]. The isolating effect of these integrated barriers has been reported to delay the release of the second biological factor by 3–70 days [35,36,37] or reduce the percentage of growth factor release without changing the temporal pattern of release [38]. A comparable approach to the 2-zone PEM film architecture in the present study has been used by Shah and coworkers employing up to 120 tetralayers containing rhBMP2 covered by up to 80 tetralayers containing rhVEGF_165_. Poly-aminoester had been used as polycation combined with poly-acrylic acid as polyanion for the BMP containing lower zone and condroitinsulfate for the VEGF-loaded upper layers. This had resulted in a slightly delayed release of rhBMP2 from the lower layers compared to the immediate release of VEGF from the upper zone [27]. In the present study, the isolating effect of a second zone of only 10 bilayers of PLL-Hep on top of the growth factor-loaded zone of 10 bilayers below has been suitable to suppress the release from the lower zone for at least 3 weeks. One possible reason for the long-lasting retarding effect of this rather low number of overlying double layers may be the fact that heparin has a higher specific affinity for growth factors than many other anionic partners in PEMs and is a strong poly-electrolyte pairing partner for PLL, which reduces the diffusivity of PEMs thereby decreasing the release from deeper layers of the constructed PEM film [39].

The quantitative in vivo results have shown that an effect of the bioactive coating on bone regeneration was not visible when the total volume of the trephine defect was considered. However, when the three peri-implant zones were analyzed separately, it became obvious that significant differences occurred in the zone immediately adjacent to the implant surface indicating that the range of growth factors released from the implant surface was limited to a distance of approximately 100 μm in the present model. Out of the experimental surfaces, only those implants with an early osteogenic characteristic have shown a significantly increased formation of new bone compared to the uncoated/unloaded titanium controls in this zone, whereas surfaces with an early angiogenic release profile did not significantly increase bone formation in the immediate peri-implant zone. When the analysis was focused even more to the central section of the implant, surfaces with rhBMP2 loading alone and those with the 2-zone architecture with rhBMP2 in the upper coating zone showed significantly increased bone formation that was even significantly higher than the uncoated Ti surface loaded with rhBMP2 and rhVEGF_165_.

The expression of CD31 as a measure for angiogenic activity in the present study has shown a differential pattern of biological response to the in vitro assessed release profile of both growth factors. At the early interval of 4 weeks, there was no significant difference in CD31 expression between the response to the loaded respective growth factors on PLL-Hep-coated surfaces vs. the bare Ti surfaces. Both loading approaches have shown a significant increase over the respective control surfaces albeit a differential effect of the retarded release from the PLL-Hep-coated surfaces compared to the bare Ti surfaces which has not been appreciable at this time. This was different in the 13-week interval, where the CD31 expression in the peri-implant tissues was significantly decreased in the groups of bare Ti surfaces to the level of the Ti control surface, indicating that the biological effect of this loading approach has ceased. In the groups of PLL-Hep-coated surfaces, distinct differences could be seen depending on the architecture and growth factor loading of the poly-electrolyte films. The surfaces with an early BMP2 release from the top layers of the 2-zone architecture or with a continuous release of BMP2 with or without simultaneous VEGF165 release maintained a level in CD31 expression after 13 weeks that was not significant from that after 4 weeks. In contrast, surfaces with early VEGF release from the top layers of the 2-zone architecture or sustained VEGF-only release have shown a significant decrease in the levels of peri-implant CD31 expression from 4 to 13 weeks that nevertheless were still significantly higher than the unloaded PLL-Hep control surfaces. Moreover, looking at the BMP2-releasing surfaces, a differential effect of the early BMP2 release from the upper zone of the 2-zone architecture with VEGF165 in the lower zone was visible with a significantly higher level of CD31 expression compared to surfaces with simultaneous BMP2/VEGF165 release and the 2-zone architecture with early release of VEGF165 from the upper zone.

These findings indicate, on the one hand, that the biological activity of the growth factors released from the PLL-Hep multilayer films in the present study has been substantially sustained compared to the adsorptive coating of the bare Ti surfaces for at least 3 months. On the other hand, they suggest that an early and continued release of BMP2 supported by a later release of VEGF165 leads to a higher level of angiogenic activity than a simultaneous release of both growth factors or an early release of VEGF with or without a later release of BMP2. This increased expression of CD31 is paralleled by a significantly increased bone volume in the group with early BMP release.

Looking at the sequence of angiogenic and osteogenic activities, the results of the present study appear to be in contradiction to the common appreciation that angiogenesis would precede osteogenic activity in order to provide inducible perivascular cells that subsequently undergo osteogenic differentiation under the influence of ostegenic signaling. The present results suggest that an initial osteogenic impulse through a pleiotrophic growth factor such as BMP2 [40,41] is more advantageous by providing a “regeneratome” [42] that is then supported by increased angiogenic signaling after a period of at least three weeks. It is interesting to note in this respect that the PEM coating with the early BMP release from the upper zone and later release of VEGF from underneath has been the only one that has consistently shown a significant correlation between the area of newly formed bone, bone–implant contact, and expression of CD31 which may indicate a more coherent process of angiogenesis, bone formation, and remodeling. However, the molecular mechanisms behind the observed effects remain largely unclear and future studies need to unravel that by including more key markers of angiogenesis and osteogenesis.

The activity of the released growth factors in the zone immediately adjacent to the surface is reflected also in the bone–implant contact rate where all three BMP-releasing surfaces had shown a significantly increased percentage of surface area covered with bone. The confined three-dimensional range of the released growth factor has been confirmed in a recent experimental study in mini pigs, where PEM film-coated polymer scaffolds loaded with much higher amounts of rhBMP2 induced bone formation only in the immediate vicinity of the scaffold [43] without irregular bone formation outside the scaffold area. The same held true when ectopic bone formation was induced [27] in PEM-coated polymer scaffolds loaded with comparable amounts of rhBMP2 and rhVEGF_165_, where bone formation was limited to the scaffold surface in a rodent model.

The generally rather low rate of bone formation in the present study compared to previous reports [27,43] may be accounted for by the experimental model where a trephine defect of 5 mm diameter is used creating an initially void space that gradually fills up with blood. Other than in three-dimensional scaffolds, biologically active signals are here presented on a two-dimensional surface with access to precursor cells originating from surrounding bone tissue that is up to 2 mm apart from the surface. Given the fact, that the range of released signals is not far beyond 100 μm, the conditions for a specific biological response would be best in the sections of the implant surface that are in contact with the walls of the trephine defect. As a consequence, the formation of new bone would likely originate from the defect wall propagating along the implant surface rather than being induced simultaneously in multiple spots across the surface. This is confirmed by the morphologic pattern of bone formation visible in the micrographs of the 13-week specimens.

The mini pig has been chosen as the experimental model species as it is considered to compare well to human conditions with respect to bone structure as well as bone remodeling and growth rate [31]. The shape of the implants used for the experiments, however, differs strongly from the commercially used screw-shaped implants and therefore the results cannot be transferred directly into the clinical situation. In particular, the large distance between the surrounding bone and the implant surface is not met in a clinical setting where implants are inserted into a precisely prepared implant bed that provides intimate contact of the surface with the adjacent bone. The distance between the bone surface and the implant surface, however, has been deliberately chosen to allow for the observation of bone regeneration across this distance and to detect effects of the various growth factor patterns released from the implant surface. Moreover, it should be kept in mind that endosseous implants are subject to loading in a clinical setting, which may well alter the condition at the bone–implant interface over time through load-induced bone remodeling independently from the growth factors released during the healing period.

## 5. Conclusions

In conclusion, the results indicate that the spatial range of released growth factors (1.5–3 μg/mL within 3 weeks) in the present model is limited to a distance of approximately 100 μm leading to an accelerating effect on osteoconductive bone formation propagating along the implant surface from the defect walls. With respect to dual growth factor release, the sequence of early release of BMP2 followed by VEGF165 appeared to best promote peri-implant bone formation and peri-implant angiogenesis, which is in contrast to the current understanding of the temporal patterns of growth factor release for enhancement of bone formation.

## Figures and Tables

**Table 1:** Experimental surface designs and growth factor loading (rhBMP2/rhVEGF165).

	rhBMP2 (μg/cm^2^)		rhVEGF_165_ (μg/cm^2^)	
	Mean	SD	Mean	SD
PLL-HEP_10_/BMP-2&PLL-HEP_10_/VEGF	3.8	1.3	5.5	0.4
PLL-HEP_10_/VEGF&PLL-HEP_10_/BMP-2	5.9	0.6	5.0	0.7
PLL-HEP_20_/VEGF + BMP-2	3.5	1.3	4.5	2.1
PLL-HEP_20_/VEGF			4.4	2.0
PLL-HEP_20_/BMP-2	5.7	0.4		
PLL-HEP_20_				
Ti/VEGF + BMP-2	2.9	1.8	4.6	0.4
Ti/VEGF			4.9	1.9
Ti/BMP-2	3.1	2.2		
Ti Control				

**Table 2:** Growth factor release (rhBMP2/rhVEGF_165_) after 21 days.

	r hBMP2 (μg/cm^2^)		rhVEGF_165_ (μg/cm^2^)	
	Mean	SD	Mean	SD
PLL-HEP_10_/BMP-2&PLL-HEP_10_/VEGF	0.06	0.01	2.63	0.27
PLL-HEP_10_/VEGF&PLL-HEP_10_/BMP-2	3.03	0.47	0.01	0.00
PLL-HEP_20_/VEGF + BMP-2	1.96	0.28	1.49	0.29
PLL-HEP_20_/VEGF			1.70	0.28
PLL-HEP_20_/BMP-2	2.59	0.43		
PLL-HEP_20_				
Ti/VEGF + BMP-2	0.89	0.66	0.16	0.05
Ti/VEGF			0.27	0.02
Ti/BMP-2	0.36	0.03		

**Table 3:** *p*-values Spearman rank correlation tests. Bold values indicate significant difference.

	Bone Area Trephine Defect vs. Bone–Implant Contact 4 Weeks	Bone Area Surface Layer vs. Bone–Implant Contact 4 Weeks	Bone Area Trephine Defect vs. Bone–Implant Contact 13 Weeks	Bone Area Surface Layer vs. Bone–Implant Contact 13 Weeks	Bone Area Surface Layer vs. CD31 Expression 4 Weeks	Bone Area Surface Layer vs. CD31 Expression 13 Weeks
PLL-HEP_10_/BMP-2 and PLL-HEP_10_/VEGF	0.266	0.329	0.397	0.266	0.544	0.397
PLL-HEP_10_/VEGF andPLL-HEP_10_/BMP-2	0.787	0.787	**0.005**	**0.019**	**0.042**	**0.019**
PLL-HEP_20_/VEGF +BMP-2	0.466	0.148	**0.019**	0.072	0.461	0.111
PLL-HEP_20_/VEGF	0.285	**0.037**	0.468	**0.001**	0.391	0.257
PLL-HEP_20_/BMP-2	0.623	**0.042**	0.704	**0.042**	0.266	0.266
PLL-HEP_20_	**0.042**	0.738	0.072	0.072	0.076	0.072
Ti/VEGF + BMP-2	0.872	0.329	0.156	**0.042**	0.266	0.787
Ti/VEGF	0.329	**0.019**	0.544	**0.019**	0.957	0.957
Ti/BMP-2	0.623	**0.005**	0.787	0.397	0.707	0.468
Ti Control	0.329	0.329	0.872	0.072	0.468	0.266

## Data Availability

Data supporting the reported results are available as Excel files upon request at schliephake.henning@med.uni-goettingen.de.

## Abbreviations

The following abbreviations are used in this manuscript:

rhBMP2	recombinant human bone morphogenic protein 2
rhVEGF165	recombinant human vascular endothelial growth factor 165
PEM	poly-electrolyte multilayer
PLL	poly-L-lysine
